# Supplementary material for: High-Glucose-Induced Metabolic and Redox Alterations Are Distinctly Modulated by Various Antidiabetic Agents and Interventions Against FABP5/7, MITF and ANGPTL4 in Melanoma A375 Cells
Source: Int J Mol Sci. 2025 Jan 24;26(3):1014. doi: 10.3390/ijms26031014 (PMC11817646; doi:10.3390/ijms26031014)
Supplement: Supplementary file 1 [file ijms-26-01014-s001.zip › ijms-3421686-supplementary.pdf]

Supplementary File
Table S1

PGC1α	Taqman	GAGTCTGTTATGGAGTGACATCG	TGTCTGTATCCAAGTCGTTCAC	/56-FAM/ACCAGCCTC/ZEN/TTTGCCCAGATCTTC/3IABkFQ/	NM_013261	1-2
HIF1α	Taqman	CCGTCATCTGTTAGCACCAT	GCTCACCATCAGTTATTTACGTG	/56-FAM/TCTAGACCA/ZEN/CCGGCATCCAGAAGT/3IABkFQ/	NM_010431	2-3
SMAD3	Taqman	TCTACCAGTTGACCCGAATG	GTGCAGCTCAATCCAGCA	/56-FAM/TCTGTCTCC/ZEN/TGTACTCCGCTCCC/3IABkFQ/	NM_005903	11-12
TRIB3	Taqman	GGCACTGAGTATACCTGCAAG	GAGTGAAAAAGGCGTAGAGGA	/56-FAM/ACAAGCATG/ZEN/TGGCTCGGCC/3IABkFQ/	NM_021158	2-3
ANPTNL4	Taqman	CCACCGACCTCCCGTTA	TTGTGGAAGAGTTGCTGGAT	*56-FAM/TGAGTTGTG/ZEN/TCTGCAGGCTGTGA/3IABkFQ/*	NM_139314	1-2
RPLP0 (36B4)	Taqman	TGTCTGCTCCCAC	TCGTCTTTAAACCCTGCGTG	56-FAM/CCCTGTCTT/ZEN/CCCTGGGCATCAC/3IABkFQ/	NM_001002	*2-3*
